# Endothelial dysfunction is associated with reduced myocardial mechano-energetic efficiency in drug-naïve hypertensive individuals

**DOI:** 10.1007/s11739-023-03402-9

**Published:** 2023-09-27

**Authors:** Chiara M. A. Cefalo, Alessia Riccio, Teresa Vanessa Fiorentino, Mariangela Rubino, Gaia Chiara Mannino, Elena Succurro, Maria Perticone, Angela Sciacqua, Francesco Andreozzi, Giorgio Sesti

**Affiliations:** 1 Department of Clinical and Molecular Medicine, University of Rome-Sapienza, 00189 Rome, Italy; 2 Department of Medical and Surgical Sciences, University Magna Graecia of Catanzaro, 88100 Catanzaro, Italy

**Keywords:** Endothelial dysfunction, Myocardial mechano-energetic efficiency, Heart failure, Cardiovascular diseases, Impaired glucose tolerance

## Abstract

Impaired myocardial mechano-energetics efficiency (MEE) was shown to predict incident heart failure, but pathophysiological mechanisms linking impaired MEE with heart failure have not been elucidated. Endothelial dysfunction is a plausible candidate because it has been associated with heart failure. This study aims to investigate the association between MEE and endothelium‐dependent vasodilation, among drug-naïve hypertensive individuals. 198 Drug-naïve hypertensive individuals participating in the CATAnzaro MEtabolic RIsk factors (CATAMERI) study were included. All participants underwent to an oral glucose tolerance test and to an echocardiogram for myocardial LVM-normalized mechano-energetic efficiency (MEEi) measurement. Endothelial-dependent and endothelial-independent vasodilatation were measured by strain-gauge plethysmography during intra-arterial infusion of acetylcholine and sodium nitroprusside, respectively. A multivariate linear regression analysis was conducted to investigate the independent association between maximal endothelial-dependent vasodilation and MEEi. Maximal ACh-stimulated forearm blood flow (FBF) was associated to decreased myocardial MEEi (*β* = 0.205, *p* = 0.002) independently of well‐established cardiovascular risk factors including age, sex, BMI, waist circumference, smoking status, total and HDL cholesterol, triglycerides, hsCRP, glucose tolerance status, and HOMA-IR index of insulin resistance. Conversely, no association was observed between SNP-stimulated vasodilation and MEEi. Endothelium-mediated vasodilation may contribute to reduce myocardial MEEi independently of several potential confounders. Because diminished myocardial MEE has been previously associated with incident heart failure, a non-invasive assessment of myocardial MEEi may improve the identification of individuals at higher cardiovascular risk who may benefit from the initiation of pharmacological treatments ameliorating the endothelial dysfunction.

**Supplementary Information:**

The online version contains supplementary material available at 10.1007/s11739-023-03402-9.

## Introduction

Growing evidence has shown that alterations in myocardial energetics characterized by a higher consumption of oxygen, and diminished left ventricular (LV) mechanical efficiency are implicated in the development of cardiovascular disease [1–8]. Cardiac work depends on the energy achieved nearly wholly by aerobic oxidation, and needs of a coupling between myocardial oxygen consumption and left ventricular (LV) function [8]. Of the entire energy produced by oxidative metabolism, the proportion utilized for contraction is about 25% in healthy individuals, whereas the remaining energy is dissipated as heat [8]. The myocardial mechano-energetic efficiency (MEE) is defined as the ratio between the LV work and the whole energy consumption corresponding to myocardial oxygen consumption (MVO_2_) [3, 9]. Precise measurements of MVO_2_ needs invasive techniques such as coronary sinus catheterization [10] or procedures expensive and time-consuming such as non-invasive positron emission tomography [11], two methods are not feasible in large-scale observational studies based on routine examinations. Recently, a non-invasive, ultrasound-based technique to measure myocardial MEE has been validated by calculating the ratio between stroke work, assessed as stroke volume × systolic blood pressure (SBP), and myocardial oxygen consumption (MVO2) estimated by the so-called double product, i.e., SBP multiplied by heart rate (HR) [2, 3, 9, 12, 13], normalized to LVM in order to obtain an estimate the amount of blood ejected in 1 s by each gram of LVM (i.e. indexed MEE, MEEi, ml/s per g) [12, 14]. This index has been associated with insulin resistance, obesity and dysglycemia [14–20], and has been found to predict incident heart failure independently of traditional cardiovascular risk factors [2, 5].

Pathophysiological mechanisms linking impaired MEE with heart failure have not been elucidated. Amongst the other ones, endothelial dysfunction is a plausible candidate, because it has been associated with heart failure both when measured in peripheral vessels [21, 22] and in coronary epicardial vessels [23], and has been associated with incident heart failure [24, 25]. However, the impact of endothelial dysfunction on myocardial MEE has not been determined yet. In order to address this issue, we investigated the association between myocardial MEEi and endothelium‐dependent vasodilation, evaluated by the strain-gauge plethysmography in response to intra-arterial infusion of acetylcholine (ACh) among nondiabetic individuals.

## Patients and methods

The study population encompasses 198 never treated hypertensive individuals participating in the CATAnzaro MEtabolic RIsk factors (CATAMERI) study, an observational study recruiting adult Caucasian individuals screened for one or more cardio-metabolic risk factors consecutively recruited at a referral university hospital of the University “Magna Graecia” of Catanzaro as previously described in details [26–28]. The exclusion criteria included: prior history of cardiovascular disease, including heart failure, valvulopathies (i.e. mitral regurgitation, aortic stenosis, aortic stenosis and aortic regurgitation), renal or hepatic failure, acute or chronic infectious diseases, chronic gastrointestinal diseases associated with malabsorption, chronic pancreatitis, immunological disorders, history of any malignant disease, treatment with steroids, use of estroprogestins for hormonal contraception or replacement treatment, and positivity for antibodies to hepatitis C virus or hepatitis B surface antigen. After an overnight fasting, participants underwent anthropometric measurements including body mass index (BMI), waist circumference, blood pressure, and heart rate, and a venous blood sample was drawn for laboratory determinations.

Blood pressure was measured in the sitting position after 5 min of quiet rest. A minimum of three blood pressure readings were taken on three separate occasions at least 2 weeks apart. Baseline blood pressure values were the average of last two of the three consecutive measurements obtained at intervals of three minutes. According to ESC/ESH guidelines, hypertension is defined as office SBP values > _140 mmHg and/or diastolic BP (DBP) values > _90 mmHg [29].

Glucose tolerance state was defined according to the American Diabetes Association (ADA) criteria, on the basis of oral glucose tolerance test (OGTT) using both fasting and 2-h post-challenge glucose levels [30]. Participants were classified as having normal glucose tolerance (NGT) when fasting plasma glucose was < 100 mg/dL (5.5 mmol/l) and 2-h post-load glucose was < 140 mg/dL (7.77 mmol/l), isolated impaired fasting glucose (IFG) when fasting plasma glucose was 100–125 mg/dL (5.5–6.9 mmol/l), and 2-h post-load glucose was < 140 mg/dL (< 7.77 mmo/l), impaired glucose tolerance (IGT) when fasting plasma glucose was < 126 mg/dL (7 mmol/l) and 2-h post-load glucose was 140–199 mg/dL (7.77–11.0 mmol/l), and type 2 diabetes mellitus (T2DM) when either fasting plasma glucose was ≥ 126 mg/dL (≥ 7 mmol/l) or 2-h post-load glucose was ≥ 200 mg/dL (≥ 11.1mmo/l) or were taking treatment with hypoglycemic agents. Insulin resistance was estimated using the validated homeostasis model assessment (HOMA-IR) index, calculated from the fasting glucose and insulin concentrations according to the formula: insulin (µU/ml) x glucose (mmol/liter)/22.5 [31].

### Forearm blood flow and vascular function measurements

Forearm blood flow (FBF) measurements were performed at 9:00 AM after overnight fasting, with the participants lying supine in a quiet air-conditioned room (22° to 24 °C) at Catanzaro Hospital as previously described in details [32–34]. Forearm volume was determined by water displacement. Under local anesthesia, a 20-gauge polyethylene catheter (Vasculon 2; Baxter Healthcare, Deerfield, IL, USA) was introduced into the brachial artery of the nondominant arm for assessment of blood pressure (Baxter Healthcare Corp) and for drug infusion. Measurement of percent change in forearm volume was obtained by a mercury-filled silastic strain-gauge placed on the widest part of the forearm. The strain-gauge was connected to a plethysmograph (model EC-4, D.E. Hokanson, Issaquah, WA) calibrated to measure the percent change in volume. The plethysmograph in turn was connected to a chart recorder to record the FBF measurements. A cuff placed on the upper arm was inflated to 40 mmHg with a rapid cuff inflator (model E-10 Hokanson, Issaquah, WA) to occlude venous outflow from the extremity. FBF was calculated as the slope of the change in forearm volume; the mean of 3 measurements was obtained at each time point. Vascular function was assessed according to the protocol described by Panza et al. [35]. Endothelium-dependent and endothelium-independent vasodilation were assessed by a dose–response curve during intra-arterial infusions of acetylcholine (ACh) (7.5, 15, and 30 µg/mL^−1^ × min^−1^, each for 5 min) and sodium nitroprusside (SNP) (0.8, 1.6, and 3.2 µg/mL^−1^ × min^−1^, each for 5 min), respectively. ACh (Sigma, Milan, Italy) was diluted with saline, and SNP (Malesci, Florence, Italy) was diluted in 5% glucose solution and protected from light with aluminum foil. The sequence of administration of ACh and SNP was randomized to avoid any bias related to the order of drug infusion.

### Echocardiography

In all participants, echocardiogram was performed by using a VIVID-7 Pro ultrasound machine (GE Technologies, Milwaukee, WI) with an annular phased array 2.5-MHz transducer as previously described [19]. The echocardiograms were performed in the morning with the participant in supine left lateral decubitus. Measurements of interventricular septal (IVS) thickness, and left ventricular internal diameter were done at end-diastole. LV end-diastolic (LVEDV) and end-systolic volume (LVESV) were assessed according to Simpson method and indexed for body surface area (BSA) [36]. LV mass (LVM) was calculated using the Devereux formula [37] and normalized by BSA [LVMI]) [36, 37]. Stroke volume was calculated as the difference between LV end-diastolic and end-systolic volumes. Myocardial mechano-energetic efficiency is influenced by two factors: external myocardial work and myocardial oxygen consumption [1–3, 9]. External myocardial work was estimated as stroke work calculated as systolic blood pressure (SBP) x echocardiographic stroke volume (SV). Myocardial oxygen consumption was measured by the “double product” of heart rate (HR) x SBP [38, 39]. Therefore, MEE was computed as: SBP × SV/SBP × HR = SV/HR where HR were expressed in seconds (HR/60). Because MEE is highly related to LVM [36], MEE was normalized to LVM to attain a measure of the amount of blood ejected in 1 s by each gram of LVM (i.e. indexed MEE, MEEi, ml/s per g) [12, 14].

### Analytical determinations

Blood glucose, triglycerides, total and high-density lipoprotein (HDL) cholesterol levels were measured by enzymatic methods (Roche, Basel, Switzerland). High sensitivity C reactive protein (hsCRP) levels were determined by an automated instrument (CardioPhase^®^ hsCRP, Milan, Italy).

### Statistical analysis

Variables with a skewed distribution including triglycerides, HOMA-IR index, and hsCRP were natural log transformed for statistical analyses. Continuous variables are expressed as mean ± standard deviation. A *χ*2 test was used to compare categorical variables. Relationships between variables were determined by Pearson’s correlation coefficient (*r*). The independent association between endothelial-dependent vasodilation and MEEi was examined by multivariate linear regression analysis. In the multivariate linear regression analysis, data are expressed as standardized regression coefficient (*β*) and *p* value. Multicollinearity among variables included in the multiple linear regression model was excluded by the fact that the variance inflection factor was less than 2. A *p* value ≤ 0.05 was considered statistically significant. All the statistical analyses were performed by SPSS software program Version 27 for Windows (IBM Corp, Armonk, NY, USA).

## Results

The metabolic and anthropometric characteristics of study subjects are shown in Table 1. The study population includes 113 men and 85 women having a mean age of 50 ± 11 years (ranging from 20 to 75 years), and mean BMI of 28.5 ± 4.5 kg/m^2^ (ranging from 18.0 to 45.7 kg/m^2^). Of the 198 participants, 111 (56.0%) had NGT, 43 (21.7%) had isolated IFG, 4 (2.0%) had IGT, and 40 (20.2%) had type 2 diabetes. Amongst individuals with type 2 diabetes, 15 subjects were treated with metformin. Echocardiographic parameters and basal forearm blood flow for the study cohort are shown in Table 2.

**Table 1:** Anthropometric and metabolic characteristics of the study subjects

Variables	Whole study subjects
Gender (men/women)	113/85
Age (yrs)	50 ± 11
BMI (kg/m^2^)	28.5 ± 4.5
Waist circumference (cm)	97 ± 11
Smoking status (never smokers/current smokers/ex-smokers) (number)	122/33/43
Systolic blood pressure (mmHg)	145 ± 17
Diastolic blood pressure (mmHg)	91 ± 11
Total cholesterol (mg/dl)	204 ± 36
HDL (mg/dl)	49 ± 12
Triglycerides (mg/dl)	132 ± 78
Fasting Glucose (mg/dl)	103 ± 29
Fasting plasma insulin (µUI/ml)	13 ± 6
HOMA-IR index	3.5 ± 2.1
hsCRP (mg/l)	4.3 ± 3.0
Glucose tolerance status (NGT/IFG/IGT/type 2 diabetes) (number)	111/43/4/40

Data are means ± SD. *BMI* body mass index, *HDL* high-density lipoprotein, *IFG* impaired fasting glucose, *IGT* impaired glucose tolerance

**Table 2:** Left ventricular geometry, mechano-energetic performance and basal forearm blood flow of the study subjects

Variables	Means ± SD
LV end-systolic volume (mL)	40.6 ± 31.8
LV end-diastolic volume (mL)	130.9 ± 52.7
LVM (g)	228 ± 74
LVM index (g/m^2^)	123 ± 35
LV ejection fraction (%)	70 ± 9
Stroke volume (mL)	90.3 ± 28.7
Stroke work (mmHg × ml)	13,118 ± 4622
Myocardial oxygen consumption (mmHg × bpm)	10,438 ± 2046
Myocardial MEEi (mL/sec/g)	0.34 ± 0.09
Basal forearm blood flow (ml/100 ml^−1^ of tissue × min^−1^)	3.25 ± 1.97

Data are means ± SD. Comparisons between the three groups were performed using a general linear model for multiple comparison. *P* values refer to results after analyses with adjustment for age and sex. *LV* Left Ventricular, *LVM* Left ventricular mass, *MEEi* LVM-normalized mechano-energetic efficiency

### Vascular function measurements

Intra-arterial infusions of ACh induced a significant dose-dependent increase in forearm blood flow (FBF) (*p* < 0.0001). The FBF increments from basal value (3.25 ± 1.97 ml/100 ml^−1^ of tissue × min^−1^) at the three incremental doses of ACh (7.5, 15, and 30 µg/mL^−1^ × min^−1^, respectively) were 5.83 ± 1.79 (+ 80%), 8.60 ± 2.83 (+ 265%), and 13.08 ± 4.53 (+ 403%) ml/100 ml^−1^ of tissue x min^−1^, respectively. Similarly, sodium nitroprusside (SNP) infusions induced a significant dose-dependent increase in FBF (*p* < 0.0001). The FBF increments from basal value at the three incremental doses of SNP (0.8, 1.6, and 3.2 µg/mL^−1^ × min^−1^, respectively) were 2.34 ± 1.16 (+ 72%), 5.76 ± 2.16 (+ 177%), and 10.97 ± 3.75 (+ 337%) ml/100 ml^−1^ of tissue x min^−1^, respectively. For the following analysis, we used only maximal vasodilatory response to both ACh and SNP [32–34]. Intra-arterial infusion of vasoactive substances did not induce significant changes in blood pressure or heart rate values.

### Association between stimulated FBF and myocardial mechano-energetic efficiency

**Fig. 1:**
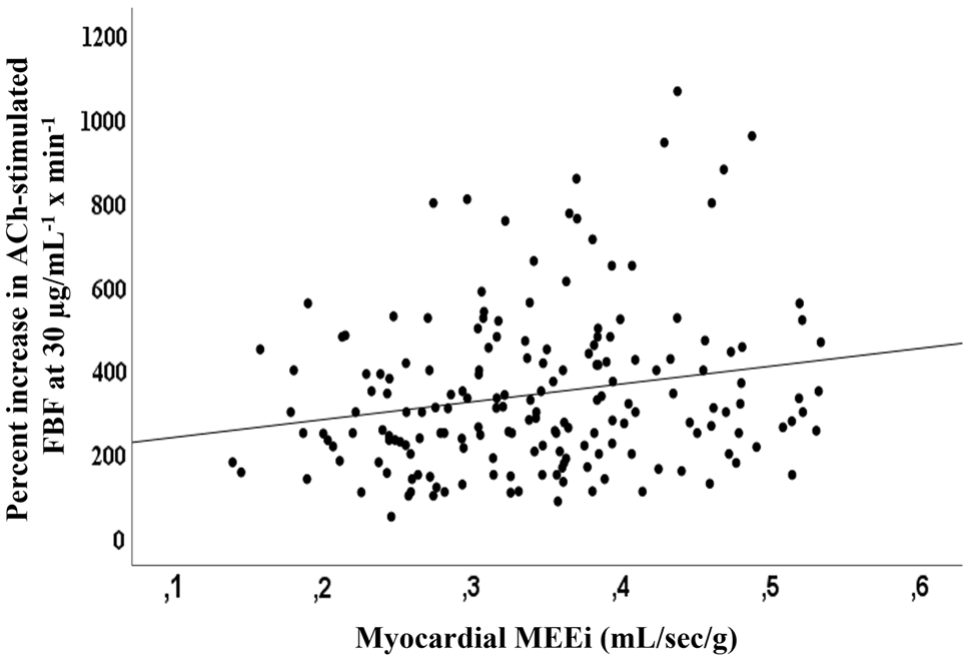
Correlation between maximal Ach-stimulated FBF and myocardial mechano-energetic efficiency

Next, to examine the association between maximal ACh-stimulated FBF and MEEi we performed a univariate analysis. Myocardial MEEi was associated with glucose tolerance status, fasting insulin, and HOMA-IR index (Table 3). Interestingly, maximal ACh-stimulated FBF was significantly associated with myocardial MEEi (Table 3, and Fig. 1). Conversely, there was no relationship between maximal SNP-stimulated vasodilation and MEEi (Table 3).

**Table 3:** Univariate correlations between myocardial MEEi, ACh-stimulated FBF, SNP-stimulated FBF, anthropometric and metabolic variables

Variables	Myocardial MEEi	
	*r*	*P*
Sex	− 0.088	0.21
Age (years)	0.042	0.55
Smoking status	0.071	0.31
BMI (kg/m^2^)	− 0.075	0.29
Waist circumference (cm)	− 0.093	0.19
Systolic blood pressure (mmHg)	− 0.122	0.08
Total cholesterol (mg/dL)	0.016	0.82
HDL-C (mg/dL)	0.011	0.88
Triglycerides (mg/dL)	− 0.006	0.93
Glucose tolerance status	− 0.146	0.04
Fasting plasma glucose (mg/dL)	0.118	0.09
Fasting insulin (µU/mL)	− 0.144	0.04
HOMA-IR index	− 0.168	0.02
hsCRP (mg/L)	− 0.005	0.94
Heart rate (bpm)	− 0.536	<0.001
Percent increase in ACh-stimulated FBF at 30 µg/mL^−1^ × min^−1^	0.215	0.002
Percent increase in SNP-stimulated FBF at 3.2 µg/mL^−1^ × min^−1^	0.099	0.25

*BMI* body mass index, *HDL-C* high-density lipoprotein-cholesterol, *HOMA-IR* homeostasis model assessment, *hsCRP* high sensitivity C reactive protein, *Ach* acetylcholine, *SNP* Sodium nitroprusside, *FBF* forearm blood flow

To estimate the independent contribution of ACh-stimulated FBF responses at 30 µg/mL− 1 × min^−1^ to myocardial MEEi, we performed a multivariate regression analysis in a model including age, gender, BMI, smoking status, total and HDL cholesterol, triglycerides, hsCRP, glucose tolerance status, and HOMA-IR index of insulin resistance. As shown in Table 4, we found that the only variable independently associated with MEEi was maximal ACh-stimulated FBF (*β* = 0.19, *p* = 0.02). Moreover, the independent association between maximal ACh-stimulated FBF and MEEi did not change when waist circumference was included in the regression model in the place of BMI values (*β* = 0.19, *p* = 0.02) (Supplementary Table 1). Additionally, the independent association between maximal ACh-stimulated FBF and MEEi did not change when systolic blood pressure was added in the regression model (*β* = 0.198, *p* = 0.02). Accordingly, maximal ACh-stimulated FBF remained significantly associated with MEEi when heart rate was further added in the regression model (*β* = 0.185, *p* = 0.02). A sensitivity analysis excluding patients with type 2 diabetes (*n* = 40) yielded an almost identical effect estimate ((*β* = 0.199, *p* = 0.012).

**Table 4:** Multiple regression analyses evaluating the association between maximal ACh-stimulated FBF, anthropometric and metabolic variables and myocardial MEEi as dependent variable

Multiple linear regression model	*R*	*R*^*2*^	*SE*	*P *value
Model 1 (age, sex, smoking status, BMI, total cholesterol, HDL, triglycerides, glucose tolerance status, HOMA-IR, hsCRP and ACh-stimulated FBF at 30 µg/mL^−1^ × min^−1^)	0.298	0.08	0.09	0.08

Covariates	Standardized Coefficient β	SE	*P* value	VIF
ACh-stimulated FBF at 30 µg/mL^−1^ × min^−1^	0.19	0.001	0.02	1.56
hsCRP (mg/l)	0.36	0.010	0.62	1.09
Glucose tolerance status (NGT/IFG/IGT/type 2 diabetes)	− 0.13	0.006	0.08	1.31
HOMA-IR index	− 0.06	0.013	0.45	1.43
Smoking status (never smokers/current smokers/ex-smokers)	0.09	0.008	0.21	1.23
Gender (men/women)	− 0.40	0.015	0.61	1.26
Age (yr)	0.12	0.001	0.10	1.37
BMI (Kg/m^2)^	− 0.03	0.002	0.97	1.19
Total cholesterol (mg/dl)	0.17	0.002	0.82	1.17
HDL (mg/dl)	0.19	0.001	0.79	1.08
Triglycerides (mg/dl)	0.03	0.001	0.65	1.18

*BMI* body mass index, *HDL-C* high-density lipoprotein-cholesterol, *hsCRP* high sensitivity C reactive protein, *Ach* acetylcholine, *FBF* forearm blood flow

## Discussion

The results of the present study demonstrate, for the first time, that myocardial MEEi, as estimated by an indirect validated method, is significantly associated with the endothelium-mediated vasodilation in a cohort of drug-naïve hypertensive individuals. In order to investigate whether endothelium-mediated vasodilation was associated with decreased myocardial MEEi independently of well-established cardio-metabolic risk factors, we performed a multivariate linear regression including several confounders such as age, sex, smoking status, BMI, total cholesterol, HDL, triglycerides, glucose tolerance status, hsCRP, and HOMA-IR index of insulin resistance. We found that maximal endothelium-mediated vasodilation was the major determinant of myocardial MEEi independently of well‐established cardiovascular risk factors known to be associated with MEEi including glucose tolerance status [15] and HOMA-IR index [14]. These data may help to shed light into the mechanism linking reduced myocardial mechano-energetic efficiency, per unit of myocardial mass, and incident heart failure [2, 40]. Prior studies have shown that endothelial dysfunction can exert an important role in the pathogenesis of heart failure [21–25] by reducing nitric oxide production, which ultimately results in vasoconstriction, pro-inflammatory and pro-oxidant state, LV systolic and diastolic dysfunction, myocardial fibrosis, and hypertrophy [41]. The present findings of an independent association between maximal endothelium-mediated vasodilation and myocardial MEEi coupled with previous studies showing the pathogenic role of impaired myocardial energetics in the development of heart failure led us to hypothesize that endothelial dysfunction may precede and induce a reduction in myocardial MEEi, which, in turn, contributes to the increased risk of heart failure [2]. Although maximal ACh-stimulated FBF explained a small proportion (4.6%) of the variability of myocardial MEEi, endothelium-mediated vasodilation remained the only cardio-metabolic variable independently associated with myocardial MEEi amongst those herein tested including age, sex, smoking status, BMI, total cholesterol, HDL, triglycerides, glucose tolerance status, hsCRP, and HOMA-IR index of insulin resistance. It is likely that other factors not measured in the present study may have had a greater impact on myocardial MEEi variability and further studies are required to address this issue.

The current results may be clinically relevant. Because diminished myocardial MEE has been associated with incident heart failure, assessment of myocardial MEEi may improve the identification of individuals at risk of heart failure requiring a closer follow-up. Moreover, these individuals may benefit from the initiation of pharmacological treatments such as ACE inhibitors and angiotensin II type 1 receptor antagonists capable to ameliorate endothelial dysfunction and, possibly, to reduce the progression to heart failure [42–51].

Pathophysiological mechanisms that may contribute to both impaired endothelium-mediated vasodilation and decreased myocardial MEEi comprise, among the others, abnormalities in body weight, glucose homeostasis, inflammation, insulin resistance, and oxidative stress [52, 53]. Notably, our finding that maximal endothelium-mediated vasodilation remained associated with myocardial MEEi even after adjustment for BMI, glucose tolerance status, hsCRP, and HOMA-IR index of insulin resistance argues against the possibility that these cardiovascular risk factors might have contributed to the observed association. Clearly, further studies are required to explore the pathophysiological mechanisms linking impaired endothelium-mediated vasodilation to depressed myocardial MEEi.

The present study has some strengths. Amongst the other ones, the inclusion of participants of both sexes with a broad spectrum of glucose tolerance. Moreover, the results are strengthened by the use of a gold standard technique for assessment of endothelium-dependent vasodilation, i.e. strain-gauge plethysmography, performed by a trained staff, following a standardized protocol, and by the measurements of hormonal and metabolic variables in fresh blood samples rather than in stored samples. Furthermore, the assessment of echocardiographic parameters was performed by a single trained examiner who was blinded to the endothelium-dependent vasodilation data of the participants. In addition, our study included only drug-naïve hypertensive individuals without the confounding effects of medical therapy, known to modulate endothelial function such as beta-blockers, angiotensin II receptor blockers, and angiotensin converting enzyme inhibitors.

Nonetheless, present study has also some potential limitations. First, our study is an observational, nonrandomized, study recruiting hypertensive outpatients at a referral university hospital, representing individuals at increased risk for cardio-metabolic disease, and, therefore, the results of this study may not be extendable to the general population. Second, the cross-sectional design of the study does not allow us to determine the causal relationship between endothelium-dependent vasodilation and changes in myocardial MEEi, and, therefore, our findings need to be confirmed in prospective observational studies. Moreover, left ventricular oxygen consumption and myocardial MEEi were assessed by indirect measures rather than invasive procedures such as coronary sinus catheterization or expensive procedures such as cardiac positron emission tomography. Nevertheless, direct assessment of myocardial energetic metabolism is unfeasible for large-scale studies. Additionally, only Caucasian subjects were recruited in the present study, and therefore, our findings cannot be generalized to other ethnic groups. Furthermore, the present results were obtained in drug-naïve hypertensive individuals, and, therefore, should be validated in subjects without hypertension. Finally, although we found that the association between endothelium-dependent vasodilation and myocardial MEEi was independent of various covariates including adiposity, lipid profile, inflammatory marker, glucose tolerance status, and insulin resistance, we cannot rule out that residual undetermined confounding factors may have affected the results.

## Conclusion and perspectives

The present study demonstrates that maximal endothelium-dependent vasodilation is independently associated with reduced myocardial mechano-energetic efficiency in a cohort of drug-naïve hypertensive individuals. Therefore, a non-invasive assessment of myocardial MEEi may contribute to reduce heart failure progression by improving the identification of individuals at higher cardiovascular risk who may benefit from the initiation of pharmacological treatments ameliorating the endothelial dysfunction. Our work supports an early endothelial dysfunction as a possible pathogenetic mechanism linking impaired MEEi to an increased incidence of heart failure in high cardiovascular risk population. Therefore, the assessment of myocardial MEEi, especially in newly diagnosticated hypertensive patients without prior cardiovascular diseases, may improve the identification of those individuals may benefit from the initiation of pharmacological treatments targeted at the nitric oxide pathway to ameliorate endothelial dysfunction and possibly prevent heart failure. However, further studies are required, firstly to validate our findings also in subjects without hypertension and secondly to explore the pathophysiological mechanisms linking impaired endothelium-mediated vasodilation to depressed myocardial MEEi.

### Supplementary Information

Below is the link to the electronic supplementary material.

Supplementary file1 (DOCX 15 KB)

## Data Availability

Raw data supporting the finding of this study are available from the corresponding author, CMAC, upon reasonable request.
