# Supplementary material for: Endothelial dysfunction is associated with reduced myocardial mechano-energetic efficiency in drug-naïve hypertensive individuals
Source: Intern Emerg Med. 2023 Sep 27;18(8):2223–30. doi: 10.1007/s11739-023-03402-9 (PMC10635990; doi:10.1007/s11739-023-03402-9)
Supplement: Supplementary file 1 — Supplementary file1 (DOCX 15 KB) [file 11739_2023_3402_MOESM1_ESM.docx]

**Supplementary Table 1 — Multiple regression analyses evaluating the association between maximal ACh-stimulated FBF, anthropometric and metabolic variables and myocardial MEEi as dependent variable.** HDL-C: high-density lipoprotein- cholesterol, hsCRP: high sensitivity C reactive protein, Ach: acetylcholine, FBF: forearm blood flow.

| **Multiple linear regression model** | ***R*** | ***R2*** | ***SE*** | ***P value*** |
| --- | --- | --- | --- | --- |
| **Model 2:** age, sex, smoking status, waist circumference, total cholesterol, HDL, triglycerides, glucose tolerance status, HOMA-IR, hsCRP and ACh-stimulated FBF at 30 µg/mL^-1^ x min^-1^ | 0.298 | 0.08 | 0.09 | 0.08 |
| **Covariates** | **Standardized Coefficient β** | **SE** | ***P value*** | **VIF** |
| ACh-stimulated FBF at 30 µg/mL^-1^ x min^-1^ | 0.19 | 0.001 | **0.02** | 1.53 |
| hsCRP (mg/l) | 0.03 | 0.010 | 0.62 | 1.08 |
| Glucose tolerance status  (NGT/IFG/IGT/type 2 diabetes) | -0.13 | 0.006 | 0.08 | 1.31 |
| HOMA-IR index | -0.61 | 0.013 | 0.46 | 1.45 |
| Smoking status  (never smokers/current smokers/ex-smokers) | 0.09 | 0.008 | 0.21 | 1.22 |
| Gender (men/women) | -0.03 | 0.015 | 0.62 | 1.35 |
| Age (yr) | 0.12 | 0.001 | 0.10 | 1.25 |
| Waist circumference (cm) | -0.005 | 0.001 | 0.94 | 1.16 |
| Total cholesterol (mg/dl) | 0.01 | 0.001 | 0.82 | 1.05 |
| HDL (mg/dl) | -0.01 | 0.001 | 0.79 | 1.18 |
| Triglycerides (mg/dl) | 0.33 | 0.001 | 0.65 | 1.16 |
